# The impact of cardiopulmonary bypass time on the Sequential Organ Failure Assessment score after cardiac surgery

**DOI:** 10.1093/icvts/ivae082

**Published:** 2024-04-29

**Authors:** Tiago R Velho, Rafael Maniés Pereira, Nuno Carvalho Guerra, Ricardo Ferreira, Dora Pedroso, Ana Neves-Costa, Ângelo Nobre, Luís Ferreira Moita

**Affiliations:** Innate Immunity and Inflammation Laboratory, Instituto Gulbenkian de Ciência, Oeiras, Portugal; Department of Cardiothoracic Surgery, Hospital de Santa Maria, Centro Hospitalar Lisboa Norte, Lisbon, Portugal; Cardiothoracic Surgery Research Unit, Centro Cardiovascular da Universidade de Lisboa (CCUL@RISE), Faculdade de Medicina da Universidade de Lisboa, Lisbon, Portugal; Department of Cardiothoracic Surgery, Hospital de Santa Maria, Centro Hospitalar Lisboa Norte, Lisbon, Portugal; Escola Superior Saúde da Cruz Vermelha Portuguesa, Lisbon, Portugal; Department of Cardiothoracic Surgery, Hospital de Santa Maria, Centro Hospitalar Lisboa Norte, Lisbon, Portugal; Department of Cardiothoracic Surgery, Hospital de Santa Maria, Centro Hospitalar Lisboa Norte, Lisbon, Portugal; Cardiothoracic Surgery Research Unit, Centro Cardiovascular da Universidade de Lisboa (CCUL@RISE), Faculdade de Medicina da Universidade de Lisboa, Lisbon, Portugal; Innate Immunity and Inflammation Laboratory, Instituto Gulbenkian de Ciência, Oeiras, Portugal; Innate Immunity and Inflammation Laboratory, Instituto Gulbenkian de Ciência, Oeiras, Portugal; Department of Cardiothoracic Surgery, Hospital de Santa Maria, Centro Hospitalar Lisboa Norte, Lisbon, Portugal; Cardiothoracic Surgery Research Unit, Centro Cardiovascular da Universidade de Lisboa (CCUL@RISE), Faculdade de Medicina da Universidade de Lisboa, Lisbon, Portugal; Innate Immunity and Inflammation Laboratory, Instituto Gulbenkian de Ciência, Oeiras, Portugal; Faculdade de Medicina da Universidade de Lisboa, Lisbon, Portugal

**Keywords:** cardiac surgery, cardiopulmonary bypass, organ dysfunction, SOFA score

## Abstract

**OBJECTIVES:**

Postoperative organ dysfunction is common after cardiac surgery, particularly when cardiopulmonary bypass (CPB) is used. The Sequential Organ Failure Assessment (SOFA) score is validated to predict morbidity and mortality in cardiac surgery. However, the impact of CPB duration on postoperative SOFA remains unclear.

**METHODS:**

This is a retrospective study. Categorical values are presented as percentages. The comparison of SOFA groups utilized the Kruskal–Wallis chi-squared test, complemented by ad hoc Dunn’s test with Bonferroni correction. Multinomial logistics regressions were employed to evaluate the relationship between CPB time and SOFA.

**RESULTS:**

A total of 1032 patients were included. CPB time was independently associated with higher postoperative SOFA scores at 24 h. CPB time was significantly higher in patients with SOFA 4–5 (**P = 0.0022) or higher (***P < 0.001) when compared to SOFA 0–1. The percentage of patients with no/mild dysfunction decreased with longer periods of CPB, down to 0% for CPB time >180min (50% of the patients with >180m in of CPB presented SOFA ≥ 10). The same trend is observed for each of the SOFA variables, with higher impact in the cardiovascular and renal systems. Severe dysfunction occurs especially >200 min of CPB (cardiovascular system >100 min; other systems mainly >200 min).

**CONCLUSIONS:**

CPB time may predict the probability of postoperative SOFA categories. Patients with extended CPB durations exhibited higher SOFA scores (overall and for each variable) at 24 h, with higher proportion of moderate and severe dysfunction with increasing times of CPB.

## Graphical abstract

**Figure:**
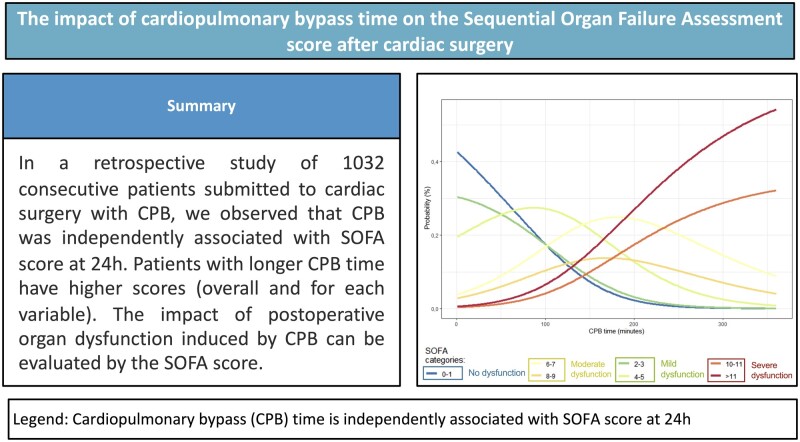

## INTRODUCTION

Postoperative organ dysfunction (POD) remains a significant challenge in cardiac surgery (CS), affecting up to 40% of patients [1]. This morbidity is intertwined with a systemic inflammatory response and several other biological processes, including ischaemia–reperfusion, oxidative stress, endothelial dysfunction and microvascular thrombosis [2]. These factors, in conjunction patient comorbidities, perioperative variables (e.g. mean arterial pressure, myocardial protection) and surgical manipulation, collectively contribute to the onset of end-organ failure [3, 4].

The prevalence and patterns of organ dysfunction following CS have not been adequately and consistently characterized. The Sequential Organ Failure Assessment (SOFA) score, a six-system measure (respiratory, cardiovascular, hepatic, coagulation, renal and neurological systems), daily assesses multiple organ failure in the intensive care unit (ICU) [5]. Initially designed for evaluating organ failure in sepsis, the SOFA score examines how interventions like the initiation of vasopressors or mechanical ventilation could impact the progression of organ dysfunction. SOFA has been employed to predict mortality and has been validated in various ICU populations [6, 7]. It has also been validated after CS, providing a reliable tool for predicting the degree of POD [5, 8].

The SOFA score holds the advantage of being significantly simpler compared to other scores commonly utilized in the ICU setting, and its application has become widespread in cardiovascular ICUs. While studies have confirmed that cardiopulmonary bypass (CPB) and aortic cross-clamp times are associated with an increased risk of POD [9–11], the specific influence of CPB on the SOFA score and its impact on each of the 6 organ systems has not been thoroughly explored [5, 12]. The primary objective of this study was to describe POS associated with CPB using the SOFA score, aiming to assess the CPB impact on both the overall score and separately on each of the 6 evaluated organ systems.

## METHODS

### Study population

The study was approved by the local ethics committee (Comissão de Ética Centro Hospitalar Lisboa Norte, Ref. No. 386/21, approved on 17 March 2022) and followed the Strengthening the Reporting of Observational Studies in Epidemiology guidelines.

This single-center retrospective study included consecutive patients submitted to CS with CPB between 1 January 2017 and 31 December 2019. The study encompassed various procedures, including valve replacement or repair, coronary artery bypass graft, ascending and aortic arch surgery and/or combined surgery. Excluding criteria comprised patients who (i) were transferred to other ICUs after surgery and (ii) did not have SOFA score calculated during ICU stay. No intermediate care unit was available and patients were directly transferred from the ICU to the cardiothoracic surgical ward. Information was sourced from our institution’s registry database, supplemented by medical records.

### Perioperative characteristics

Preoperative variables, including past medical history and comorbidities, along with operative variables, were retrospectively collected from the clinical files from our department. EuroSCORE II assessments were conducted preoperatively for each patient, as previously published [13].

Surgical procedures adhered to standardized protocols based on the specific type of surgery. Heparin (300 mg/kg) was administered to achieve an activated clotting time >480 s. Non-pulsatile roller pump was used with blood flow indexed to 2.4 l/min/m^2^. Intermittent antegrade cold blood cardioplegia was used for induction and warm for reperfusion. Most surgeries were performed with mild hypothermia to normothermia (target 32–36°C), monitored through a nasopharyngeal probe. Heparin was reversed with protamine (1:1 according to the used heparin dose). Blood glucose levels were maintained below 250 mg/dl and minimal allowable haematocrit was 24%. Vasopressors were initiated in case of persistent hypotension. In valvular procedures, the choice of heart prostheses was determined based on the preferences of both the patient and the surgeon.

### Sequential Organ Failure Assessment *calculation*

The SOFA score was calculated in the ICU every 24 h, commencing on the first postoperative day, as previously described, until discharge [14]. In this study, we focused on the SOFA score calculated on the first postoperative day (SOFA score at 24 h). SOFA was calculated considering the variables previously published (Supplementary Material, Table S1) [7], assessing the degree of dysfunction of 6 organ systems (respiratory, cardiovascular, hepatic, coagulation, renal and neurological), scoring each from 0 (no dysfunction) to 4 (severe dysfunction) points. The assumed Glasgow Coma Scale values were used in sedated patients until demonstrated otherwise [10].

For classification purposes, we categorized no organ dysfunction as an overall score of 0, mild POD with a score between 1 and 3, moderate POD with a score between 4 and 9 and severe POD with a score of 10 or more, considering the assumptions outlined in the published works that were instrumental in developing the SOFA score [7, 15–17]. For each of the systems within the SOFA score, we considered 0 as no dysfunction, 1 as mild POD, 2 as moderate POD and 3 and 4 as severe POD [7, 15–17].

### Statistics

Continuous variables are presented as means and standard deviation for normally distributed values or as median with interquartile ranges for non-normal distributions. Categorical variables are expressed as percentages.

To evaluate the relationship between the categorical variable ‘cardiopulmonary bypass time’ and the organ systems included in the SOFA score, we employed a Kruskal–Wallis test followed by a multiple comparison test (Dunn’s test). Subsequently, to determine which groups differed from each other, we performed a multiple comparison test using the Dunn test with Bonferroni correction.

We performed several multinomial logistic models (1 for each SOFA category) to explore the association between the dependent variables (respiratory, coagulation, hepatic, cardiovascular, renal, neurologic) and the independent variables ‘age’, ‘sex’, ‘procedures’, ‘surgery on aorta’ and ‘cardiopulmonary bypass time’. The model was adjusted using the multinom() function from the Exact statistical package in R. The dependent variables represent the response categories of the variable, while the independent variables encompass demographic information (age and sex) and surgical variables (procedures, surgery on aorta and CPB time).

Moreover, the same model was adjusted for the dependent variable related to the outcome of the SOFA score, incorporating the same significant variables. This adjustment aimed to investigate the probabilities of each category based on the explanatory variables. Prior to conducting the multinomial analysis, all model assumptions were scrutinized to ensure the validity of the results and the appropriateness of the model. Specific analyses were performed for each assumption, including diagnostic plots, multicollinearity tests and other relevant methods, with the goal of confirming the suitability of the multinomial model for the analysed data.

All statistical tests conducted are 2 sided, and *P*-values <0.05 were considered statistically significant. Statistical analyses were performed using R, version 4.2.1 (R Foundation for Statistical Computing, Vienna, Austria).

## RESULTS

### Patient demographic data

We enrolled a total of 1032 patients submitted to CS with CPB and were subsequently admitted to the ICU. Supplementary Material, Table S2 provides a comprehensive overview of demographic data. Most patients were submitted to elective surgery, with 65.5% (650 patients) undergoing non-coronary artery bypass graft procedures, 28.5% undergoing 2 procedures and 5.9% undergoing 3 or more procedures. Thoracic aortic surgery was performed in 12.4% of cases.

### Cardiopulmonary bypass and postoperative organ dysfunction

**Figure 1:**
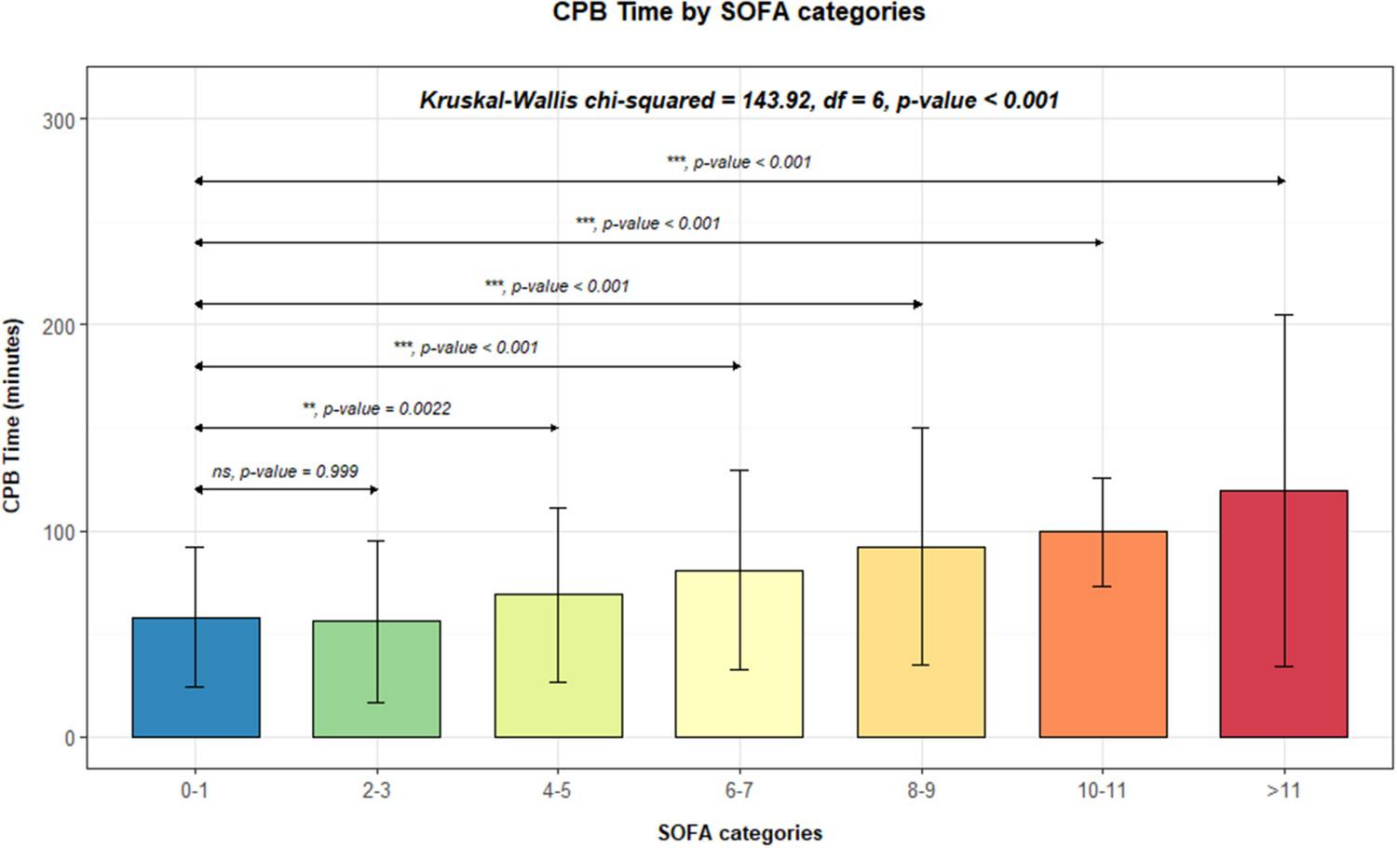
Median cardiopulmonary bypass time according to SOFA score categories. SOFA 0–1 category served as the reference group for comparison with other categories, using a Kruskal–Wallis chi-squared test. The following symbols were used in figures to indicate statistical significance: ns: non-significant; *P* < 0.05 (*); *P* < 0.01 (**); *P* < 0.001 (***); *P* < 0.0001 (****). SOFA: Sequential Organ Failure Assessment.

Considering all patients, only 177 (17.2%) exhibited no organ dysfunction (overall SOFA score of 0). Then, we decided to investigate the relation between the SOFA score of all patients 24 h after surgery and CPB time. The analysis revealed that longer periods of CPB heightened the likelihood of higher postoperative SOFA scores at 24 h. Moreover, patients with higher SOFA scores and more severe organ dysfunction demonstrated significantly longer median CPB time, as illustrated in Fig. 1. Using a Kruskal–Wallis chi-squared test, complemented by ad hoc Dunn’s test with Bonferroni correction, we confirmed that the median CPB time was markedly higher in patients with SOFA score 4–5 (**P = 0.0022) or higher (***P < 0.001), in comparison to those with SOFA scores of 0–1.

**Figure 2:**
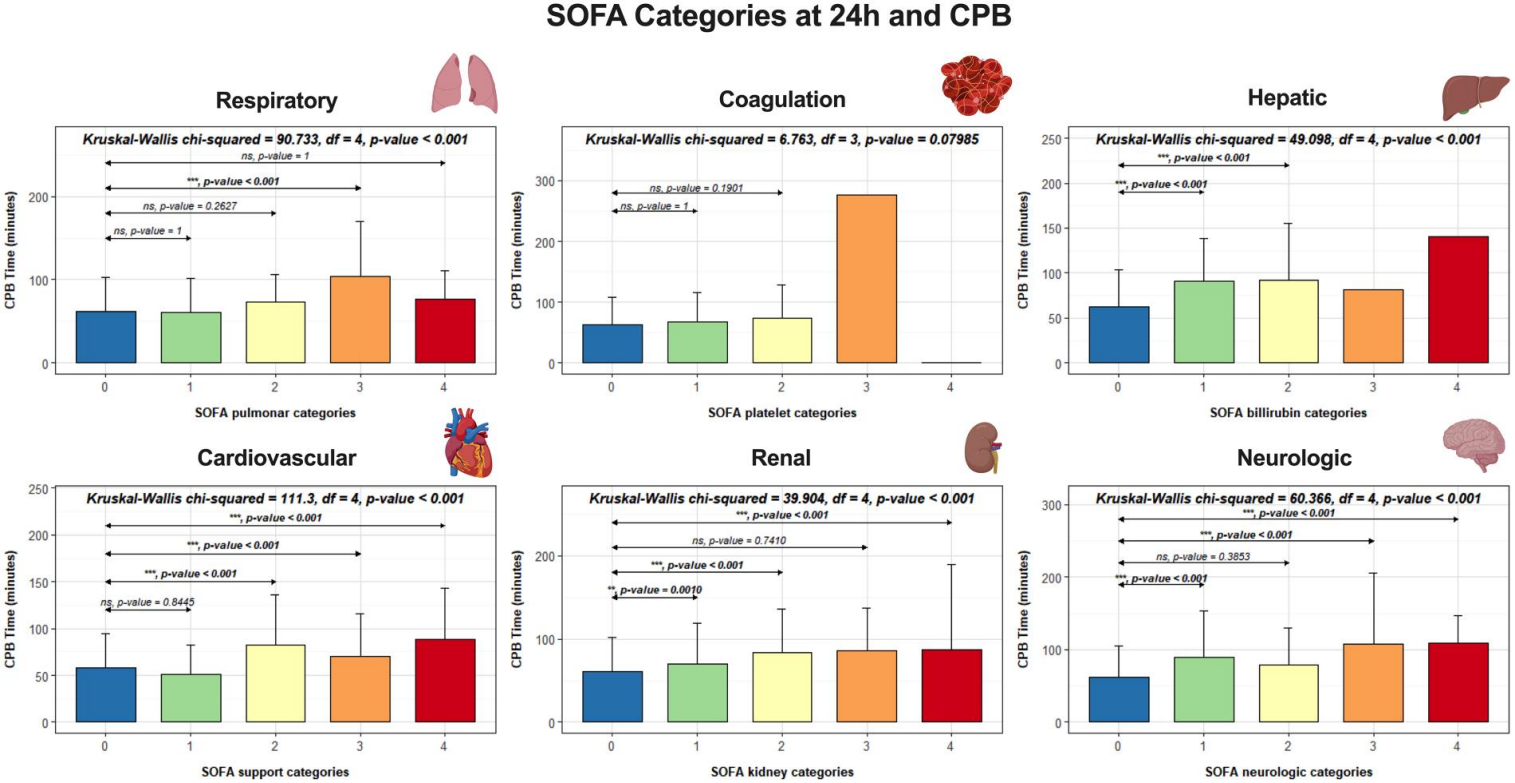
Median cardiopulmonary bypass time for all scores of each system of the SOFA score. For each system, SOFA 0 was used as the reference group for comparison with other scores, using a Kruskal–Wallis chi-squared test. The following symbols were used in figures to indicate statistical significance: ns: non-significant; *P* < 0.05 (*); *P* < 0.01 (**); *P* < 0.001 (***); *P* < 0.0001 (****). SOFA: Sequential Organ Failure Assessment.

To further explore the association of CPB time with each of the 6 systems, we calculated the median CPB time for each variable (Fig. 2). In the coagulation and hepatic systems, only 1 patient presented a score of 3 or 4, 24 h after surgery, making multiple comparisons in these 2 systems inappropriate. Utilizing a Kruskal–Wallis test, we observed that, beside the coagulation system, there were statistically significant differences in median CPB time between all scores (from 0 to 4) for each SOFA score system. Subsequently, for each SOFA system, we compared the median CPB times of patients with a score of 0 (no dysfunction) with each of the other scores (ranging from 1 to 4), using Dunn’s test with subsequent Bonferroni correction for *P*-values. Patients who presented no organ dysfunction (SOFA score 0) exhibited considerably lower median CPB times compared to higher SOFA scores, particularly scores of 3 or 4, which demonstrated higher median CPB times.

**Figure 3:**
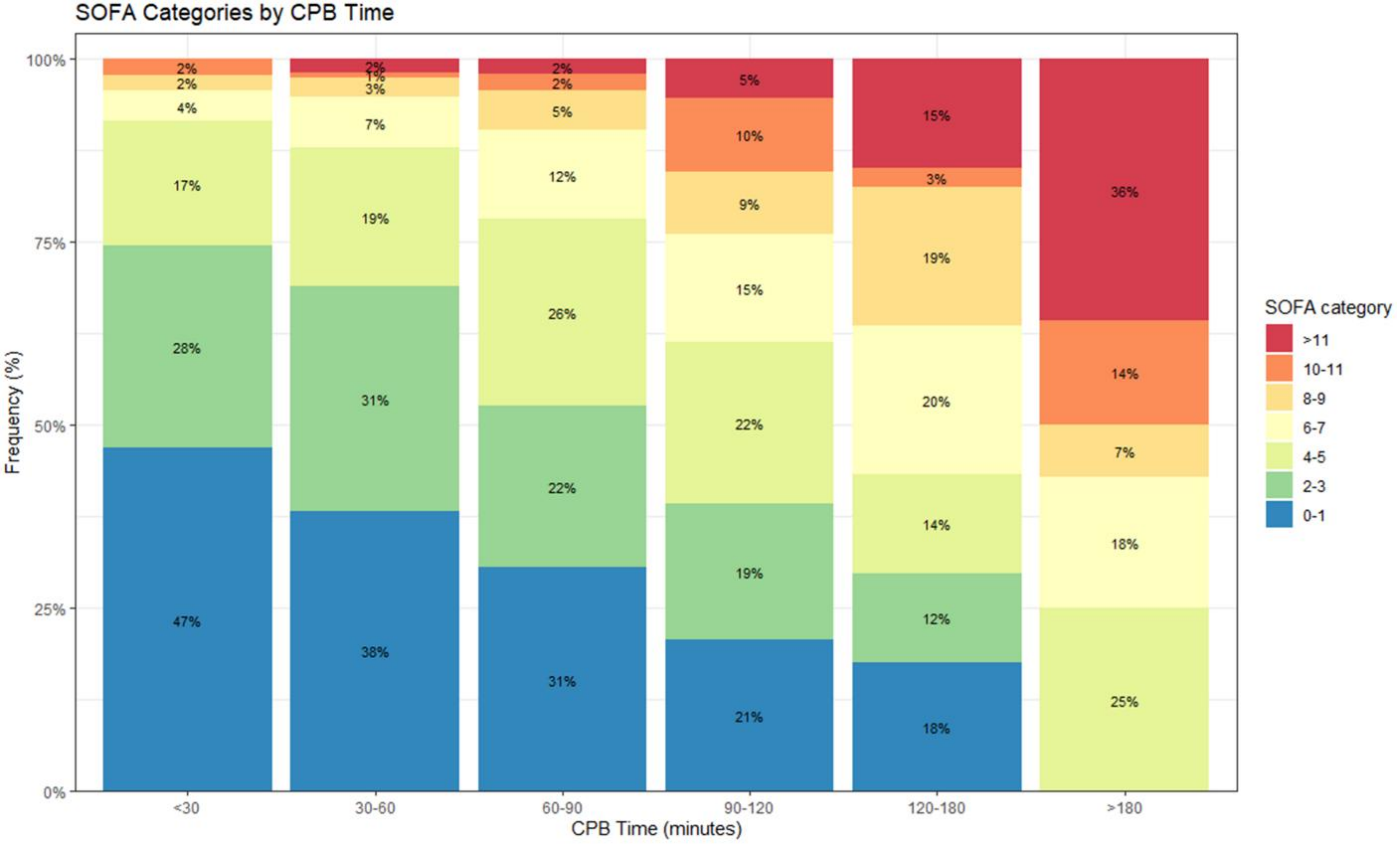
Proportion (in percentage, %) of patients with different SOFA score categories according to cardiopulmonary bypass time. No organ dysfunction or mild perturbation was considered with SOFA up to 3; moderate organ dysfunction with SOFA between 4 and 9; and severe dysfunction with a SOFA score of at least 10. SOFA: Sequential Organ Failure Assessment.

In our sample, the proportion of patients experiencing either no POD or only mild perturbations decreased with longer periods of CPB (Fig. 3). None of the patients with CPB <30 min exhibited SOFA scores above 11. For patients with CPB duration ranging between 30 and 60 min, the proportion with no dysfunction or mild perturbation was 69%, with only 3% presenting severe dysfunction scores. In parallel, there was a noticeable rise in the proportion of patients displaying moderate and severe organ dysfunction 24 h after surgery. Intriguingly, none of the patients with CPB duration above 180 min presented with no or mild POD, with 50% of this subgroup presenting a SOFA score of at least 10, indicative of severe dysfunction (Fig. 3). Therefore, an increase in CPB time appears to be associated with a higher probability of POD, as assessed by the SOFA score at 24 h, a relationship that we intend to explore more comprehensively in the future.

**Figure 4:**
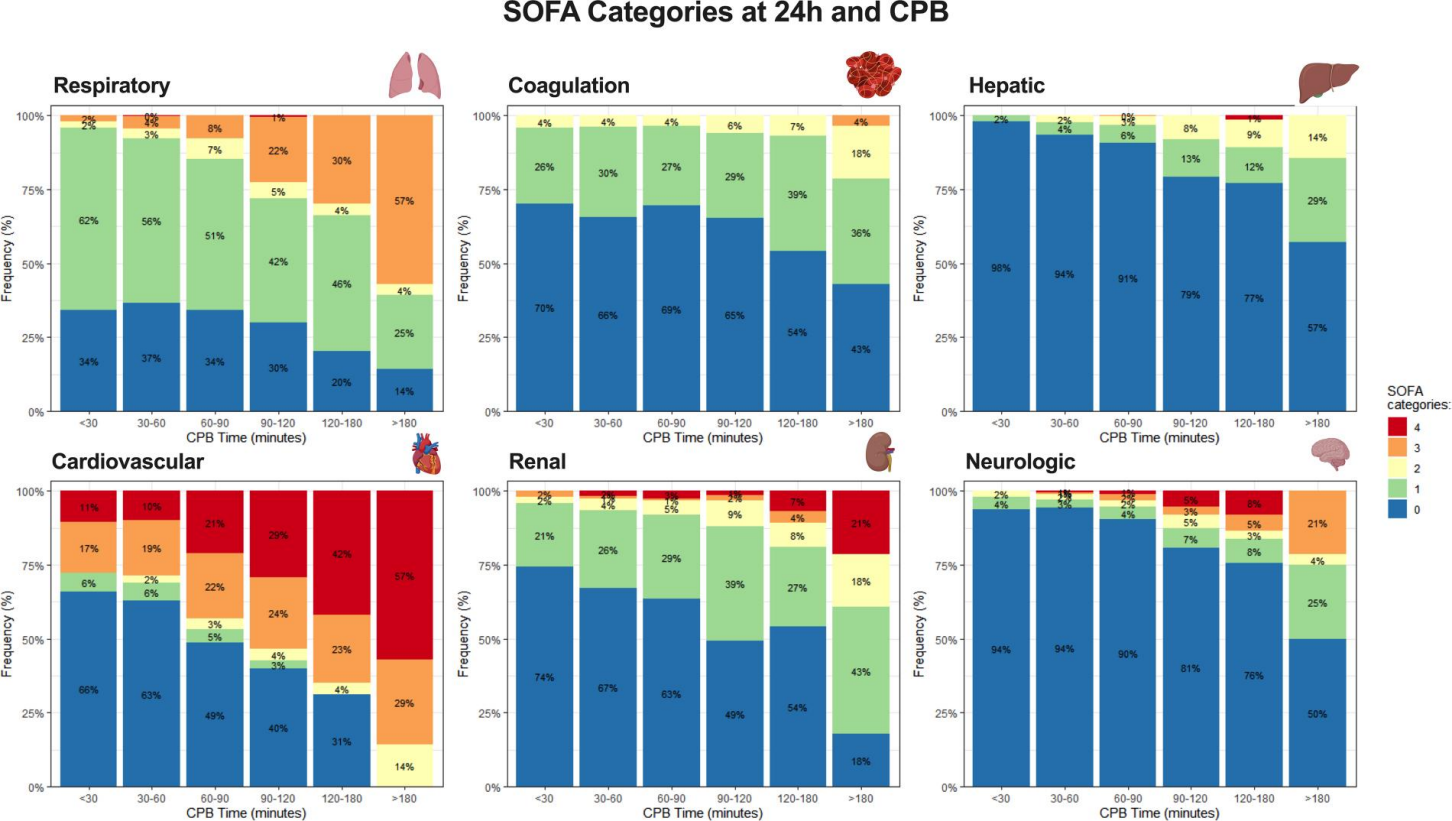
Proportion (in percentage, %) of patients with different SOFA score categories according to cardiopulmonary bypass time for each of the systems. No organ dysfunction or mild perturbation was considered with SOFA up to 3; moderate organ dysfunction with SOFA between 4 and 9; and severe dysfunction with a SOFA score of at least 10. SOFA: Sequential Organ Failure Assessment.

We subsequently examined whether the observed trend extended to each of the individual organ systems comprising the SOFA score (Fig. 4). The results indicated an association between CPB time and the severity of organ dysfunction across all 6 variables. In each category, prolonged CPB duration was linked to reduced proportions of patients experiencing no or mild organ dysfunction. Notably, the impact of CPB time was more pronounced in the cardiovascular and renal systems (Fig. 4).

### Cardiopulmonary bypass as a predictor of postoperative organ dysfunction

To better understand how CPB impacts the SOFA score in comparison to other variables such as age, type of procedure performed and thoracic aorta surgery, we employed a multinomial logistic regression with SOFA 0–1 as the reference category (Table 1). Compared to the reference category, CPB time emerged as an independent factor associated with a higher SOFA score, particularly from SOFA 4–5 (***P < 0.001). As expected, age also exhibits a significant impact across all groups, with higher ages correlating with higher probabilities of increased POD as indicated by an elevated SOFA score. Female sex showed a statistically significant lower chance of having moderate POD with SOFA 2–3, compared to 0–1. The same effect was observed for severe dysfunction with SOFA > 11. Regarding the type of procedure, the performance of 3 or more procedures only had a significant impact on moderate to severe organ dysfunction, likely attributed to the inherent increase in CPB time associated with more complex procedures.

**Table 1:** Multinomial logistic regression analysis of relevant variables to each category of Sequential Organ Failure Assessment score

SOFA score	2–3			4–5			6–7			8–9			10–11			>11		
Characteristic	OR	95% CI	*P*-value	OR	95% CI	*P*-value	OR	95% CI	*P*-value	OR	95% CI	*P*-value	OR	95% CI	*P*-value	OR	95% CI	*P*-value
Age	1.05	1.03–1.07	<0.001	1.03	1.01–1.04	0.005	1.06	1.03–1.08	<0.001	1.03	1.00–1.06	0.033	1.07	1.02–1.11	0.002	1.04	1.00–1.07	0.026
Sex																		
Male																		
Female	0.60	0.42–0.85	0.004	1.00	0.70–1.44	>0.9	0.69	0.43–1.10	0.12	0.65	0.35–1.20	0.2	0.78	0.36–1.70	0.5	0.31	0.14–0.71	0.005
Procedure																		
Single non-CABG																		
2 procedures	1.08	0.72–1.63	0.7	0.95	0.62–1.46	0.8	1.27	0.76–2.12	0.4	2.01	1.03–3.90	0.040	0.87	0.38–2.01	0.7	2.09	0.95–4.60	0.066
≥3 procedures	1.41	0.41–4.88	0.6	3.80	1.34–10.8	0.012	3.27	1.04–10.3	0.043	6.96	2.04–23.8	0.002	1.15	0.20–6.80	0.9	5.71	1.51–21.6	0.010
Thoracic aorta	0.90	0.50–1.64	0.7	1.00	0.56–1.77	>0.9	1.23	0.64–2.38	0.5	0.56	0.21–1.46	0.2	2.23	0.90–5.49	0.082	1.54	0.67–3.57	0.3
CPB	1.00	1.00–1.01	0.3	1.01	1.01–1.02	<0.001	1.02	1.02–1.03	<0.001	1.02	1.02–1.03	<0.001	1.03	1.02–1.04	<0.001	1.04	1.03–1.04	<0.001

CABG: coronary artery bypass grafting; CI: confidence interval; CPB: cardiopulmonary bypass; OR: odds ratio; SOFA: Sequential Organ Failure Assessment.

**Figure 5:**
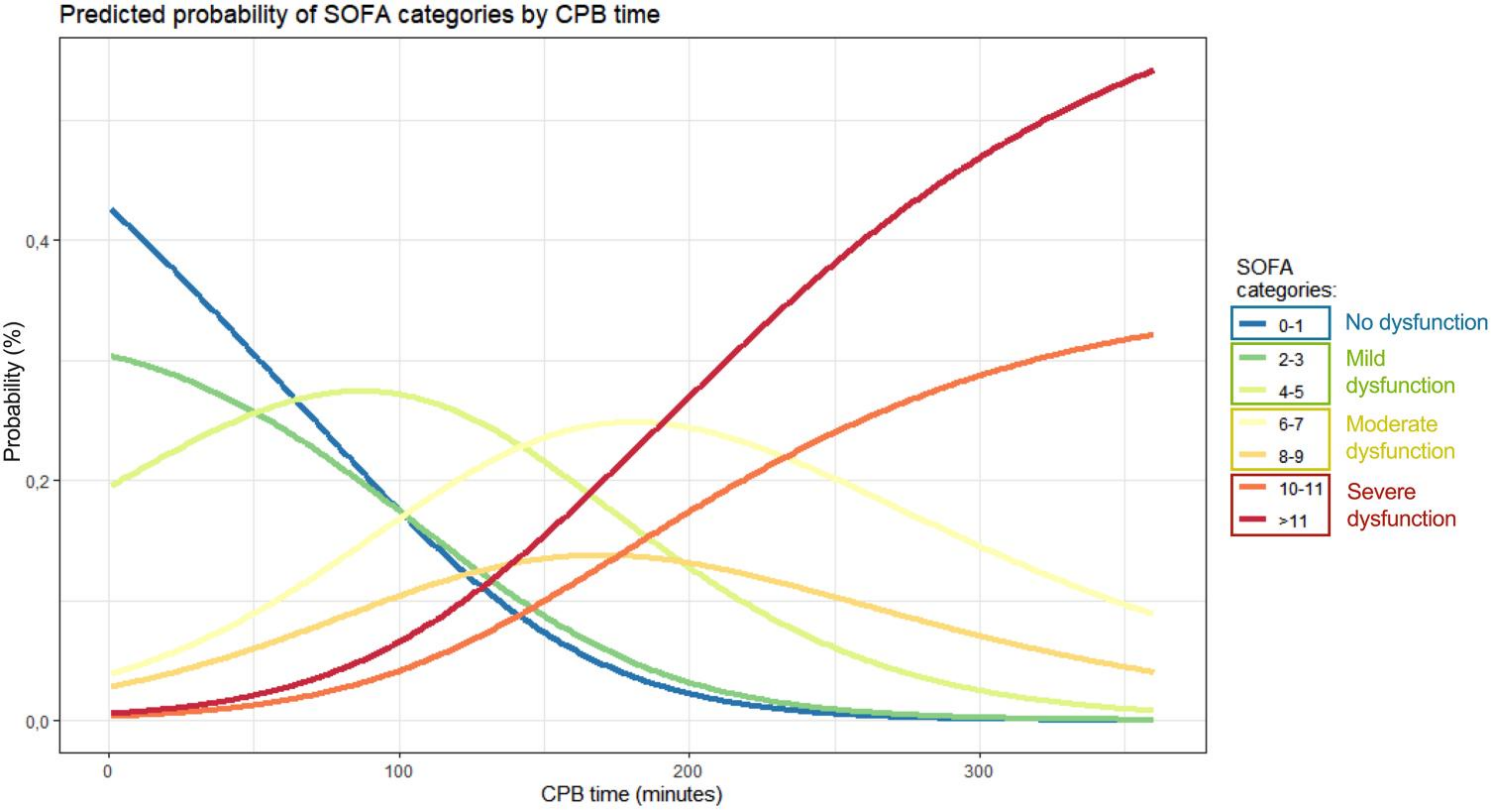
Predicted probability (in percentage, %) for each of the SOFA categories according to cardiopulmonary bypass time. No organ dysfunction or mild perturbation was considered with SOFA up to 3; moderate organ dysfunction with SOFA between 4 and 9; and severe dysfunction with a SOFA score of at least 10. SOFA: Sequential Organ Failure Assessment.

After constructing our model, we calculated the predicted probability of falling in one of the SOFA score categories based on CPB time (Fig. 5). Figure 5 illustrates the probability of a patient falling into a particular SOFA category according to CPB time, assuming that age corresponds to the median of the sample. The probability of experiencing no POD or only mild perturbations decreased with longer periods of CPB, dropping abruptly until around 200 min of CPB, when it approached 0%. With 100 min of CPB, the probability of having no organ dysfunction or only mild perturbation (SOFA 0–1 and 2–3) was ∼40%, with a predicted probability of severe POD around 10%. Simultaneously, the probability of severe dysfunction scores increased with CPB time, and CPB durations over 200 min were associated with nearly 0% probability of having no organ dysfunction or only mild perturbation. Categories associated with moderate organ dysfunction displayed a more consistent pattern up to 150 min of CPB, after which they decreased, giving way to an exponential rise in the probability of severe organ dysfunction (SOFA 10–11 and >11). Severe organ dysfunction became even more prevalent beyond 200 min of CPB, with an almost 50% probability of having a SOFA score of at least 10 and a probability of no POD or only mild perturbation approaching 0%.

We employed the same methodology to understand the impact of CPB and other pertinent variables on each of the systems incorporated in the SOFA score (Table 2). Using SOFA score 0 (no organ dysfunction) as the reference category, we found that, except for the pulmonary system, CPB time was independently associated with an increased likelihood of higher SOFA scores across various systems. In the pulmonary system, higher values of SOFA appeared to be less dependent on CPB time (only statistically significant for a score of 3). However, thoracic aorta surgery was independently associated with SOFA scores of 3 and 4 in the pulmonary system (Table 2). Interestingly, age was not associated with an increased risk of higher SOFA scores in the cardiovascular system, being only significant for a score of 4 (Table 2). This observation aligns with our previous findings that the impact of CPB is more pronounced and relevant in the cardiovascular system.

**Table 2:** Multinomial logistic regression analysis of relevant variables to each organ system of Sequential Organ Failure Assessment score

SOFA score	Characteristic	Respiratory			Coagulation			Hepatic			Cardiovascular			Renal			Neurologic		
		OR	95% CI	*P*-value	OR	95% CI	*P*-value	OR	95% CI	*P*-value	OR	95% CI	*P*-value	OR	95% CI	*P*-value	OR	95% CI	*P*-value
1	Age	1.01	1.00–1.03	0.049	1.03	1.01–1.04	<0.001	1.01	0.98–1.03	0.5	1.02	0.98–1.05	0.3	1.06	1.04–1.08	<0.001	1.04	1.01–1.08	0.012
	Sex																		
	Male																		
	Female	0.86	0.64–1.14	0.3	0.62	0.46–0.83	0.001	0.77	0.46–1.29	0.3	1.50	0.81–2.79	0.2	0.52	0.39–0.71	<0.001	1.15	0.63–2.09	0.6
	Procedure																		
	Single non-CABG																		
	2 procedures	0.81	0.58–1.12	0.2	0.80	0.58–1.11	0.2	1.42	0.83–2.46	0.2	2.14	1.09–4.21	0.027	1.24	0.89–1.73	0.2	1.10	0.57–2.13	0.8
	≥3 procedures	0.66	0.34–1.30	0.2	0.79	0.42–1.47	0.5	1.68	0.72–3.94	0.2	0.00	0.00–0.00	<0.001	1.35	0.72–2.53	0.4	0.63	0.20–2.05	0.4
	Thoracic aorta	0.93	0.59–1.46	0.7	0.77	0.49–1.21	0.3	0.74	0.36–1.52	0.4	1.57	0.60–4.12	0.4	1.13	0.72–1.77	0.6	0.48	0.17–1.33	0.2
	CPB	1.00	1.00–1.01	0.2	1.01	1.00–1.01	0.002	1.01	1.01–1.02	<0.001	0.99	0.97–1.00	0.076	1.01	1.01–1.02	<0.001	1.02	1.01–1.03	<0.001
2	Age	1.01	0.98–1.04	0.6	1.07	1.03–1.11	<0.001	1.00	0.97–1.03	0.9	1.01	0.98–1.05	0.5	1.02	1.00–1.05	0.11	1.00	0.97–1.05	0.8
	Sex																		
	Male																		
	Female	0.75	0.40–1.41	0.4	0.89	0.48–1.65	0.7	0.98	0.51–1.90	>0.9	1.02	0.49–2.15	>0.9	0.39	0.20–0.73	0.003	0.45	0.18–1.15	0.095
	Procedure																		
	Single non-CABG																		
	2 procedures	1.04	0.52–2.08	>0.9	0.59	0.28–1.24	0.2	2.10	1.02–4.33	0.045	0.84	0.36–1.95	0.7	1.26	0.67–2.37	0.5	1.98	0.78–5.00	0.15
	≥3 procedures	0.76	0.19–2.99	0.7	1.42	0.53–3.76	0.5	3.50	1.33–9.26	0.011	1.96	0.47–8.22	0.4	1.37	0.49–3.84	0.5	3.24	0.84–12.5	0.089
	Thoracic aorta	0.87	0.34–2.27	0.8	1.66	0.74–3.69	0.2	0.82	0.34–1.98	0.7	0.60	0.19–1.90	0.4	1.24	0.59–2.61	0.6	0.73	0.20–2.58	0.6
	CPB	1.01	1.00–1.02	0.073	1.01	1.01–1.02	<0.001	1.01	1.01–1.02	<0.001	1.02	1.01–1.03	<0.001	1.02	1.01–1.02	<0.001	1.01	0.99–1.02	0.3
3	Age	1.01	0.99–1.03	0.3							1.01	1.00–1.03	0.2	1.02	0.97–1.08	0.4	1.01	0.97–1.06	0.5
	Sex																		
	Male																		
	Female	0.74	0.45–1.19	0.2							1.26	0.90–1.77	0.2	0.09	0.01–0.72	0.023	0.64	0.25–1.62	0.3
	Procedure																		
	Single non-CABG																		
	2 procedures	1.31	0.78–2.18	0.3							1.31	0.90–1.93	0.2	1.12	0.31–4.05	0.9	1.02	0.40–2.56	>0.9
	≥3 procedures	1.28	0.56–2.92	0.6							4.91	2.20–11.0	<0.001	1.17	0.12–11.6	0.9	0.65	0.13–3.21	0.6
	Thoracic aorta	1.87	1.04–3.34	0.035							1.07	0.64–1.79	0.8	0.97	0.19–4.97	>0.9	1.82	0.69–4.79	0.2
	CPB	1.02	1.02–1.03	<0.001							1.01	1.00–1.02	<0.001	1.01	1.0–1.03	0.2	1.02	1.01–1.03	<0.001
4	Age	0.93	0.83–1.03	0.2							1.02	1.00–1.04	0.037	1.05	1.01–1.09	0.027	1.01	0.97–1.06	0.6
	Sex																		
	Male																		
	Female	1.29	0.07–22.3	0.9							1.21	0.84–1.74	0.3	0.41	0.17–0.95	0.039	0.27	0.08–0.97	0.045
	Procedure																		
	Single non-CABG																		
	2 procedures	0.00	0.00–0.00	<0.001							1.53	1.03–2.27	0.033	1.45	0.62–3.39	0.4	1.15	0.41–3.21	0.8
	≥3 procedures	30.1	0.53–1.725	0.10							3.62	1.59–8.21	0.002	1.11	0.28–4.46	0.9	1.69	0.40–7.17	0.5
	Thoracic aorta	0.00	0.00–0.00	<0.001							1.07	0.64–1.79	0.8	0.83	0.28–2.41	0.7	3.93	1.45–10.7	0.007
	CPB	0.98	0.93–1.04	0.6							1.02	1.02–1.03	<0.001	1.02	1.02–1.03	<0.001	1.01	1.00–1.02	0.009

CABG: coronary artery bypass grafting; CI: confidence interval; CPB: cardiopulmonary bypass; OR: odds ratio; SOFA: Sequential Organ Failure Assessment.

**Figure 6:**
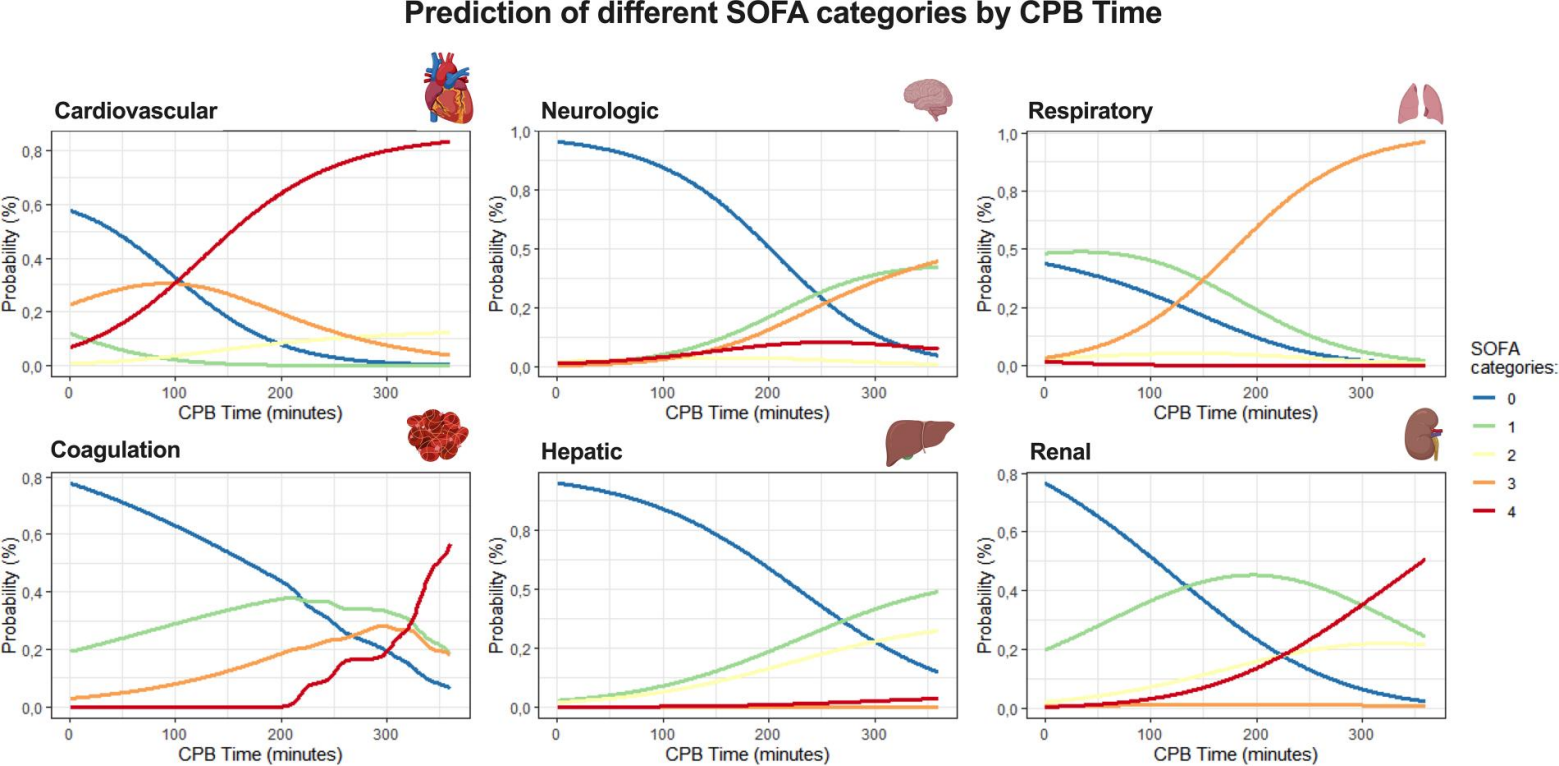
Predicted probability (in percentage %) for each of the SOFA categories according to cardiopulmonary bypass time for each of the systems included in SOFA. No organ dysfunction or mild perturbation was considered with SOFA up to 3; moderate organ dysfunction with SOFA between 4 and 9; and severe dysfunction with a SOFA score of at least 10. SOFA: Sequential Organ Failure Assessment.

The predictive probability of dysfunction for each system was calculated based on the previously described model. Figure 6 illustrates the probability of a patient with the median age of the sample having each of the scores (0 to 4) in the 6 systems, according to CPB time. The impact of CPB was notably higher in the cardiovascular system, exhibiting an exponential increase in the probability of having a higher score after 100 min of CPB. On the other hand, in the remaining systems, higher degrees of dysfunction were primarily observed after 200 min of CPB. The probability of having no dysfunction (score 0) or mild dysfunction (score 1) with 100 min of CPB was only around 30% in the cardiovascular system, compared to approximately 60% in the respiratory and 90% in the neurologic, coagulation and hepatic systems. Considering a patient with 200 min of CPB, the predicted probability of having a severe POD in the cardiovascular system was ∼85%, compared to 20% in the neurologic, 65% in the pulmonary, 20% in coagulation, 5% in the hepatic and 20% in the renal systems.

## DISCUSSION

Here, we have explored the correlation between CPB time and postoperative SOFA score, showing the accuracy of SOFA score in directly assessing and classifying CPB-related organ dysfunction. Among patients undergoing CS with CPB, a considerable proportion experienced POD at 24 h, with only 17.2% presenting without any degree of dysfunction as assessed by the SOFA score. Furthermore, our analysis revealed that CPB had a distinct impact on each of the 6 systems evaluated by the SOFA score.

When we evaluated the impact of CPB time on SOFA values in each system, we observed that the cardiovascular and renal systems were the most affected, followed by the respiratory system. This aligns with existing literature that has extensively explored the influence of CPB on the cardiovascular and renal systems, highlighting its contribution to the postoperative need for prolonged cardiovascular pharmacological support and the occurrence of acute renal injury [10, 18–20]. Importantly, our study not only reaffirms this understanding but also demonstrates that such dysfunction can be properly assessed and quantified by the use of SOFA score. Additionally, our model has also the advantage of presenting the predicted probabilities for the impact of the overall SOFA score and for each of the 6 systems, according to CPB time.

Classically, morbidity associated with CS has been predominantly attributed to the use of CPB. CPB induces a systemic inflammatory response syndrome with multifactorial contributions, including surgical trauma, ischaemia and reperfusion lesions, endothelial dysfunction, haemolysis, contact of blood with CPB artificial surfaces and activation of the coagulation cascade leading to thrombosis [2–4]. Foreign surfaces within the CPB circuit may act as triggers initiating the systemic response and sustaining the inflammatory status for a certain period, until other factors, such as aortic cross-clamp time, myocardial ischaemia and other end-organ lesions, come into play and contribute to the overall process [21, 22]. While the contact of blood with foreign surfaces appears to be a critical factor in initiating the systemic inflammatory response, the entire process remains incompletely understood [21]. It is well established that CPB duration is correlated with postoperative complications and increased length of stay in the ICU [18]. Despite significant advances in recent years, CPB remains an important source of morbidity and mortality in CS [9, 10].

POD is observed in nearly all cardiac surgeries, manifesting with variable degrees of severity [23]. Our data suggest that, for the majority of patients, organ dysfunction is an intrinsic aspect of CS, and the procedure itself imparts a distinctive organ dysfunction signature, irrespective of the diagnosis, comorbidities and surgical intervention. This signature is especially pronounced in the cardiovascular, renal and respiratory systems. Patients who experience postoperative complications not only face prolonged stays in the ICU and hospital but also endure significant morbidity extending several weeks after discharge, often necessitating readmission [24, 25]. Moreover, POD in the ICU after CS has been associated with long-term mortality at both 12 and 24 months [14].

Therefore, there is now widespread acknowledgement that morbidity stands as a major determinant of quality of care and serves as a more meaningful indicator of the success of a surgical procedure, in contrast to mortality [26, 27]. In order to properly assess morbidity, several tools have been developed to measure and evaluate the risk of postoperative complications following CS [28]. However, it is worth noting that scores used in CS exhibit a considerably lower predictive value for morbidity than for mortality [29], justifying ongoing efforts in the field. The use of more accurate scoring systems for classifying morbidity, such as the one presented in this study, is expected to contribute to more accurate patient classification. The ongoing development of improved predictive models for morbidity is a valuable pursuit, poised to enhance patient care and outcomes.

Given the widespread adoption of the SOFA score in the context of CS, it becomes crucial to understand how specific aspects of CS, such as the use of CPB, influence the overall score and each of its systems. Understanding these dynamics is essential for leveraging the SOFA score as a tool to measure, predict and subsequently reduce POD. In the current era marked by the prominence of big data and artificial intelligence (AI), our observations open the door for the implementation of more advanced models to predict POD, integrating SOFA data with other relevant clinical information. AI holds promise as a potentially more accurate tool for predicting morbidity, given the intricate and multifactorial network of events contributing and lead to POD [30]. However, the efficacy of AI is contingent on the availability of comprehensive data; thus, the establishment of detailed clinical data registries and robust clinical correlations is essential to improve the application of AI [30]. The use of SOFA score in this context not only aids in predicting organ dysfunction but also facilitates the classification of the severity of induced organ dysfunction. Moreover, it may contribute to initiating measures to anticipate and prevent further lesions.

In conclusion, our study highlights the significance of the SOFA score as a valuable tool for directly assessing and classifying CPB-related POD. To further enhance our understanding, additional studies are warranted to evaluate the predictive value of SOFA for healthcare-associated costs and quality of life across various clinical settings.

### Limitations

This study is limited by its retrospective design, limiting the strength of causal inferences. The findings, being derived from a single-centre study, the findings are applicable to the specific population under analysis, and caution should be exercised when extrapolating them to broader populations. The sample size, especially in some score comparisons, is also a limitation, preventing the execution of multiple comparisons. Furthermore, the use of a consecutive sampling strategy for patient inclusion resulted in a heterogenous population, introducing variability. The study encompasses a range of complex surgical procedures performed on patients with diverse disease severities and comorbidities, potentially influencing the duration of surgeries and CPB times, particularly in cases with more severe conditions.

Despite the meticulous adjustment of our model for various factors, including patient characteristics and surgical complexity, the inherent diversity in surgical cases requires consideration. More severe diseases and comorbidities may require longer surgeries with prolonger CPB time. While our model accounted for several factors, this inherent variability must be kept in mind.

## CONCLUSION

Our study established an association between CPB time and POD as assessed by the SOFA score. Patients undergoing longer CPB times exhibit higher SOFA scores at 24 h, and the percentage of patients without organ dysfunction or with mild perturbations decreases with increasing CPB times. CPB time is also associated with elevated SOFA scores across all 6 systems evaluated, with pronounced impacts on the cardiovascular and renal systems, followed by the respiratory system. CPB time has a predictive value for the probability of POD, classified by the SOFA score, extending to both the overall SOFA score and each of the individual organ systems.

## Supplementary Material

ivae082_Supplementary_Data

## Data Availability

The derived data generated in this research will be shared on reasonable request to the corresponding author.

## ABBREVIATIONS

AI: Artificial intelligence
CPB: Cardiopulmonary bypass
CS: Cardiac surgery
ICU: Intensive care unit
POD: Postoperative organ dysfunction
SOFA: Sequential Organ Failure Assessment
