# Supplementary material for: The impact of cardiopulmonary bypass time on the Sequential Organ Failure Assessment score after cardiac surgery
Source: Interdiscip Cardiovasc Thorac Surg. 2024 Apr 29;38(5):ivae082. doi: 10.1093/icvts/ivae082 (PMC11096272; doi:10.1093/icvts/ivae082)
Supplement: ivae082_Supplementary_Data [file ivae082_supplementary_data.docx]

**Supplementary Tables**

**Supplementary Table 1 – Sequential Organ Failure Assessment Score (SOFA)**

| **Variable** | **SOFA Score** |
| --- | --- |
| **Respiratory PaO_2_/FiO_2_ (mmHg)**  > 400  ≤ 400  ≤ 300  ≤ 200  ≤ 100 | 0  1  2  3  4 |
| **Platelets (x10^3^/μL)**  >150  ≤ 150  ≤ 100  ≤ 50  ≤ 20 | 0  1  2  3  4 |
| **Bilirrubin (mg/dL)**  <1.2  1.2-1.9  2-5.9  6-11.9  >12 | 0  1  2  3  4 |
| **Hypotension**  No hypotension  MAP < 70mmHg  Dop ≤ 5 or Dob any dose  Dop > 5, Epi ≤ 0.1 or Norepi ≤ 0.1  Dop > 15, Epi > 0.1 or Norepi > 0.1 | 0  1  2  3  4 |
| **Glasgow Coma Scale**  15  14-13  10-12  6-9  < 6 | 0  1  2  3  4 |
| **Creatinine (mg/dL) or**  **Urinary Output (mL/d)**  < 1.2  1.2-1.9  2-3.4  3.5-4.9 or < 500  > 5 or < 200 | 0  1  2  3  4 |

PaO2/ FiO2: ratio of arterial oxygen partial pressure to fractional inspired oxygen; MAP: mean arterial pressure; Dop: dopamine; Dob: dobutamine; Epi: epinephrine; Norepi: norepinephrine.

| Variable | All  Patients | SOFA  0-1 | SOFA  2-3 | SOFA  4-5 | SOFA  6-7 | SOFA  8-9 | SOFA  10-11 | SOFA  >11 |
| --- | --- | --- | --- | --- | --- | --- | --- | --- |
| N | 1032 | 316 | 251 | 218 | 112 | 58 | 33 | 44 |
| Age, years. median (IQR) | 71 (64-77) | 70 (61-76) | 73 (67-78) | 71 (65-77) | 72 (66-77) | 71 (63-77) | 72 (64-79) | 66 (59-77) |
| Male sex, n (%) | 593 (57.5) | 168 (53.2) | 153 (61) | 115 (52.8) | 68 (60.7) | 37 (63.8) | 19 (57.6) | 33 (75) |
| Hypertension, n (%) | 855 (82.8) | 255 (80.7) | 215 (85.7) | 181 (83) | 96 (85.7) | 45 (77.6) | 33 (100) | 30 (68.2) |
| Diabetes mellitus, n (%) | 298 (28.9) | 91 (28.8) | 76 (30.3) | 69 (31.7) | 33 (29.5) | 17 (29.3) | 1 (3) | 11 (25) |
| Dyslipidemia, n (%) | 616 (59.7) | 191 (60.4) | 158 (62.9) | 126 (57.8) | 71 (63.4) | 29 (50) | 17 (51.5) | 24 (54.5) |
| Chronic kidney disease, n (%) | 238 (23.1) | 49 (15.5) | 47 (18.7) | 52 (23.9) | 32 (28.6) | 23 (39.7) | 10 (30.3) | 25 (56.8) |
| Peripheral vascular disease, n (%) | 72 (6.9) | 12 (3.8) | 22 (8.8) | 15 (6.9) | 8 (7.1) | 7 (12.1) | 3 (9.1) | 5 (11.4) |
| Cerebrovascular disease, n (%) | 64 (6.2) | 11 (3.5) | 15 (6) | 17 (7.8) | 8 (7.1) | 4 (6.9) | 4 (12.1) | 5 (11.4) |
| Chronic lung disease, n (%) | 124 (12) | 34 (10.8) | 27 (10.8) | 23 (10.6) | 24 (21.4) | 7 (12.1) | 6 (18.2) | 3 (6.8) |
| Previous cardiac surgery, n (%) | 17 (1.6) | 6 (1.9) | 4 (1.6) | 2 (0.9) | 3 (2.7) | 2 (3.4) | 0 (0) | 0 (0) |
| Preserved LV function, n (%) | 892 (86.5) | 292 (92.4) | 226 (90) | 182 (83.5) | 89 (79.5) | 44 (75.9) | 29 (87.9) | 30 (68.2) |
| Moderate LV function 31-50%, n (%) | 63 (6.1) | 12 (3.8) | 14 (5.6) | 16 (7.3) | 10 (8.9) | 6 (10.3) | 3 (9.1) | 2 (4.5) |
| Poor LV function (21-30%), n (%) | 54 (5.2) | 8 (2.5) | 7 (2.8) | 16 (7.3) | 9 (8) | 6 (10.3) | 0 (0) | 8 (18.2) |
| Very Poor LV function (<20%), n (%) | 23 (2.2) | 4 (1.3) | 4 (1.6) | 4 (1.8) | 4 (3.6) | 2 (3.4) | 1 (3) | 4 (9.1) |
| Elective, n (%) | 932 (90.3) | 296 (93.7) | 235 (93.6) | 204 (93.6) | 98 (87.5) | 51 (87.9) | 19 (57.6) | 29 (65.9) |
| Urgent, n (%) | 59 (5.7) | 10 (3.2) | 13 (5.2) | 9 (4.1) | 10 (8.9) | 4 (6.9) | 7 (21.2) | 6 (13.6) |
| Emergent, n (%) | 41 (4) | 10 (3.2) | 3 (1.2) | 5 (2.3) | 4 (3.69 | 3 (5.2) | 7 (21.2) | 9 (20.5) |
| Single non-CABG, n (%) | 671 (65) | 231 (73.1) | 177 (70.5) | 142 (65.1) | 61 (54.5) | 24 (41.4) | 20 (60.6) | 16 (36.4) |
| 2 procedures, n (%) | 297 (28.8) | 80 (25.3) | 68 (27.1) | 55 (25.2) | 39 (34.8) | 24 (41.4) | 11 (33.3) | 20 (45.5) |
| 3 procedures, n (%) | 64 (6.2) | 5 (1.6) | 6 (2.4) | 21 (9.6) | 12 (10.7) | 10 (17.2) | 2 (6.1) | 8 (18.2) |
| Thoracic aorta surgery, n (%) | 135 (13.1) | 35 (11.1) | 23 (9.2) | 28 (12.8) | 19 (17) | 6 (10.3) | 11 (33.3) | 13 (29.5) |
